# A single amino acid polymorphism in the glycosyltransferase CpsK defines four *Streptococcus suis* serotypes

**DOI:** 10.1038/s41598-017-04403-3

**Published:** 2017-06-22

**Authors:** David Roy, Taryn B. T. Athey, Jean-Philippe Auger, Guillaume Goyette-Desjardins, Marie-Rose Van Calsteren, Daisuke Takamatsu, Masatoshi Okura, Sarah Teatero, Martín Alcorlo, Juan A. Hermoso, Mariela Segura, Marcelo Gottschalk, Nahuel Fittipaldi

**Affiliations:** 1 Swine and Poultry Infectious Diseases Research Centre, Faculty of Veterinary Medicine, University of Montreal, Saint-Hyacinthe, QC J2S 2M2 Canada; 2 Public Health Ontario, Toronto Laboratory, Toronto, ON M5G 1M1 Canada; 3 Saint-Hyacinthe Research and Development Centre, Agriculture and Agri-Food Canada, Saint-Hyacinthe, QC J2S 8E3 Canada; 4 Division of Bacterial and Parasitic Diseases, National Institute of Animal Health, National Agriculture and Food Research Organization, Tsukuba, Ibaraki 305-0856 Japan; 5 The United Graduate School of Veterinary Sciences, Gifu University, Gifu, Gifu 501-1193 Japan; 6 Department of Crystallography and Structural Biology, Instituto de Química-Física “Rocasolano”, Consejo Superior de Investigaciones Científicas, 28006 Madrid, Spain; 7 Department of Laboratory Medicine and Pathobiology, University of Toronto, Toronto, ON M5S 1A8 Canada

## Abstract

The capsular polysaccharide (CPS) is the major virulence factor of the emerging zoonotic pathogen *Streptococcus suis*. CPS differences are also the basis for serological differentiation of the species into 29 serotypes. Serotypes 2 and 1/2, which possess identical gene content in their *cps* loci, express CPSs that differ only by substitution of galactose (Gal) by *N*-acetylgalactosamine (GalNAc) in the CPS side chain. The same sugar substitution differentiates the CPS of serotypes 14 and 1, whose *cps* loci are also identical in gene content. Here, using mutagenesis, CPS structural analysis, and protein structure modeling, we report that a single amino acid polymorphism in the glycosyltransferase CpsK defines the enzyme substrate predilection for Gal or GalNAc and therefore determines CPS composition, structure, and strain serotype. We also show that the different CPS structures have similar antiphagocytic properties and that serotype switching has limited impact on the virulence of *S. suis*.

## Introduction

*Streptococcus suis* is a major swine pathogen and an increasingly recognized agent of zoonotic disease^1^. At least 29 *S. suis* serotypes are defined based on a serological reaction directed against the capsular polysaccharide (CPS), a crucial virulence factor with antiphagocytic properties^2–5^. Strains of serotype 2 are highly prevalent worldwide and frequently isolated from diseased swine^1^. Some serotype 2 genetic lineages such as sequence type (ST) 1, common in European and Asian countries, are highly virulent^1^. Clonal serotype 2 strains belonging to ST7, another highly virulent genotype, were responsible for two major outbreaks of *S. suis* human disease that affected hundreds of patients in China^6^. Other serotype 2 genetic lineages such as ST25 and ST28 are considered less virulent^7^, although strains belonging to both ST25 and ST28 have caused human disease^1^. Strains of serotype 14 are also often associated with zoonotic disease^1^. One recurring problem for diagnostics laboratories is that strains of zoonotic serotypes 2 and 14 cross-react in the coagglutination test (the most commonly used *S. suis* serotyping scheme) with strains of non-zoonotic serotypes 1/2 and 1, respectively^8–10^.

CPS biosynthesis in *S. suis* appears to proceed through the flippase/polymerase (Wzx/Wzy)-dependent pathway originally described for lipooligosaccharide biosynthesis^11, 12^, in which an initial monosaccharide is linked as a sugar phosphate to a membrane-associated lipid carrier by an initial sugar transferase, followed by sequential addition of sugar residues by specific glycosyltransferases. The repeating units are then translocated across the cytoplasmic membrane by Wzx, polymerized to form the lipid-linked CPS by Wzy, and finally attached to the peptidoglycan by the membrane protein complex^13^. Pioneering work by Smith *et al*. identified that all genes needed for *S. suis* serotype 2 CPS biosynthesis cluster in a single *cps* locus^11, 14^. Further studies identified *cps* loci in all other *S. suis* serotypes^15, 16^. In addition to genes encoding various different glycosyltransferases, polymerases, transferases and translocases, the *cps* loci of some serotypes also contain genes encoding additional enzymes involved in modifications of sugar residues, or in the biosynthesis and linkage of sialic acid to the CPS side chain^11, 14–16^.

**Figure 1:**
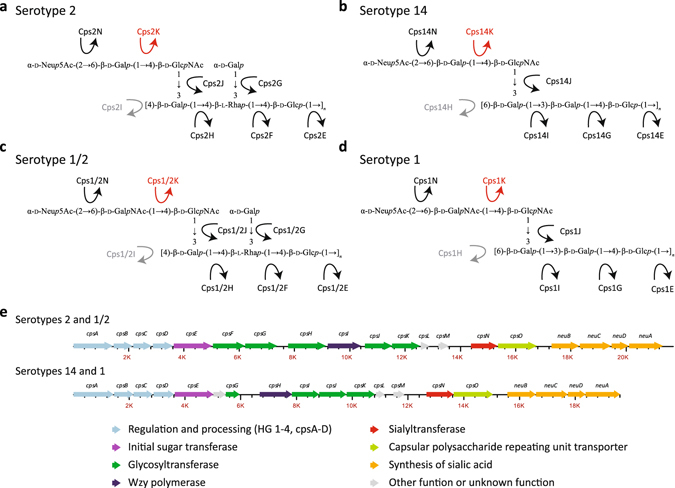
Capsular polysacharide structures of *S. suis* serotypes 2, 1/2, 14, and 1, and schematics of the *cps* loci of these serotypes. (**a**–**d**) CPS structures of serotype 2 (**a**), serotype 14 (**b**), serotype 1/2 (**c**), and serotype 1 (**d**). In serotypes 1 and 1/2, CpsK is predicted to catalyze the transfer of the side chain *N*-acetylgalactosamine (GalNAc) residue to the CPS repeating unit, while in serotypes 2 and 14, CpsK would catalyze the transfer of the galactose (Gal) residue at the same corresponding side chain. (**e**), All enzymes involved in CPS biosynthesis, including CpsK, are encoded by genes located in a single *cps* locus. Serotypes 2 and 1/2 and serotypes 14 and 1 have identical CPS gene content and organization. The putative functions of the enzymes encoded by *cps* genes are depicted with different colors. Please note that Cps enzymes (and *cps* genes) have been renamed compared to previous publications^11, 14, 16–19^ to reflect recent developments in actual or predicted function.

We have recently determined the CPS structures of serotypes 2, 1/2, 14 and 1. The serotype 2 CPS contains galactose (Gal), glucose, *N*-acetylglucosamine, rhamnose, and sialic acid^17^, while the serotype 14 CPS possesses Gal, glucose, *N*-acetylglucosamine, and sialic acid^18^. The serotype 1/2 CPS differs from the serotype 2 CPS and the serotype 1 CPS from the serotype 14 CPS by a single substitution of the Gal residue bearing the sialic acid in the serotypes 2 and 14 CPS side chains by an *N*-acetylgalactosamine (GalNAc) residue^16, 19^ (Fig. 1a–d). Interestingly, despite the aforementioned differences in CPS sugar composition and structure and the fact that all other serotypes possess a “serotype-specific” gene, serotype pairs 2 and 1/2, and 1 and 14 have identical *cps* gene content (Fig. 1e)^16^. Thus, there is no specific glycosyltransferase permitting to explain the differential addition of Gal or GalNAc to the CPS side chains of these serotypes^16^. To investigate the issue in more detail, we recently sequenced the genomes of seven strains each of serotypes 2 and 1/2, and seven strains each of serotypes 14 and 1. We found that the only consistent difference in the *cps* loci of strains of these serotype pairs was a nonsynonymous single-nucleotide polymorphism (SNP) in codon 161 of gene *cpsK*, predicted to result in a single amino acid difference in the glycosyltransferase CpsK (W161 in serotypes 2 and 14, and C161 in serotypes 1/2 and 1)^20^.

Here, we tested the hypothesis that this single amino acid polymorphism is the key factor influencing the sugar residue (Gal or GalNAc) added to the CPS repeating unit by either CpsK variants. We show that polymorphic CpsK variants define expression by *S. suis* strains of either serotype 2 or 1/2 CPSs, or either serotype 14 or 1 CPSs, and that it is possible to achieve serotype switching of field strains of serotypes 2 and 1/2, and 14 and 1 solely by replacing amino acid 161 of CpsK. We also report that serotype switching does not modify the virulence of the strains in an experimental infection model.

## Results

### Exchange of *cpsK* alleles differing by a single-nucleotide polymorphism between strains of serotypes 2 and 1/2, and between strains of serotypes 14 and 1, results in serotype switching

We hypothesized that the SNP at codon 161 of *cpsK* gene confers CpsK with different substrate predilection and results in the preferential addition of Gal (W161; serotypes 2 and 14) or GalNAc (C161; serotypes 1/2 and 1) residues to the nascent CPS repeating unit. Consequently, replacement of the W161 CpsK variant by the C161 CpsK variant, or vice versa, should result in strain serotype switching. To begin to test this hypothesis, we generated by allelic exchange the following *cpsK* isoallelic mutants: (i) strain SS2to1/2 (derived from a serotype 2 field strain, has a W161C substitution in CpsK); (ii) strain SS1/2to2 (derived from a serotype 1/2 field strain; has a C161W substitution in CpsK); (iii) strain SS14to1 (derived from a serotype 14 field strain; has a W161C substitution in CpsK), and (iv) strain SS1to14 (derived from a serotype 1 field strain; has a C161W substitution in CpsK). Whole-genome sequencing of parental and mutant strains confirmed the intended mutation, and did not identify spurious mutations elsewhere in the genome of the mutant strains, with the exception of strain SS1to14, which, compared to the WT serotype 1 strain, presented additional polymorphisms in gene *gatB*, encoding one subunit of a putative aspartyl/glutamyl-tRNA amidotransferase. These additional polymorphisms might impact the pool of arginine and glutamate amino acids of the mutant strain but are unlikely to affect CPS expression.

**Figure 2:**
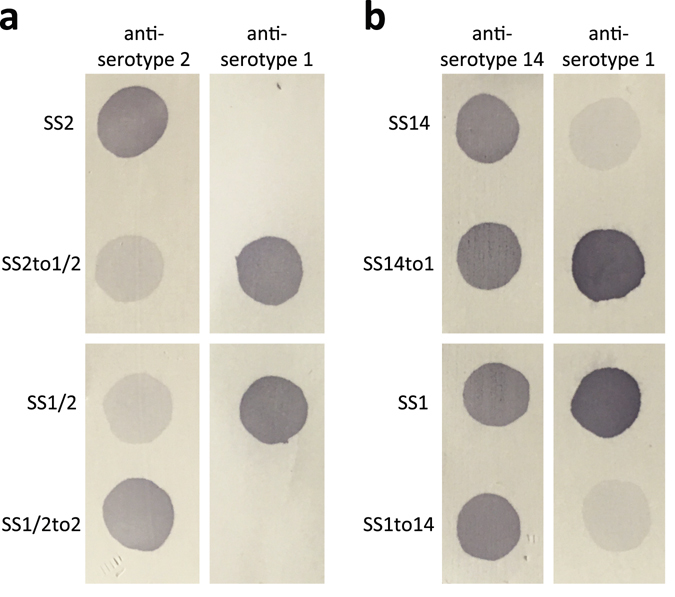
Serotype switching of mutants is confirmed by dot blotting of purified CPS preparations and specific antisera. (**a**) The CPS from a serotype 2 field strain reacts with anti-serotype 2 but not with anti-serotype 1 sera, while the CPS from mutant strain SS2to1/2 (W161C) reacts with both antisera (top panel). The CPS from a serotype 1/2 field strain reacts with both anti-serotype 1 and anti-serotype 2 sera, while the CPS from mutant strain SS1/2to2 (C161W) reacts with anti-serotype 2 but not with anti-serotype 1 sera (bottom panel). (**b**) The CPS from a serotype 14 field strain reacts strongly with anti-serotype 14 and weakly with anti-serotype 1 sera, while the CPS from mutant strain SS14to1 (W161C) reacts strongly with both antisera (top panel). The CPS from a serotype 1 field strain reacts strongly with both anti-serotype 14 and anti-serotype 1 sera, while the CPS from mutant strain SS1to14 (C161W) reacts strongly with anti-serotype 14 but weakly with anti-serotype 1 sera (bottom panel).

In all cases, parental and mutant strains expressed CPS of comparable thickness as determined by transmission electron microscopy (TEM) (Supplementary Fig. 1). When examined in the coagglutination test, all mutant strains appeared to have switched serotype (Table 1). However, since the coagglutination test uses polyclonal antibodies that may potentially recognize antigens other than the CPS, we next performed dot blotting with the same antisera and purified CPS from each pair of field and mutant strains. Consistent with the hypothesis of serotype switching, the CPS from the serotype 2 field strain reacted with anti-serotype 2 but not with anti-serotype 1 sera, while the CPS from mutant strain SS2to1/2 (W161C) reacted with both antisera (Fig. 2a, top panel). Essentially similar results were observed for CPS preparations from a serotype 14 field strain and its mutant SS14to1 (W161C) when blotted with anti-serotype 14 and anti-serotype 1 sera (Fig. 2b, top panel). As expected, the CPS from the serotype 1/2 field strain reacted with both anti-serotype 1 and anti-serotype 2 sera, while the CPS from mutant strain SS1/2to2 (C161W) reacted with anti-serotype 2 but not with anti-serotype 1 sera (Fig. 2a, bottom panel). Essentially similar results were observed for CPS preparations from the serotype 1 field strain and its mutant SS1to14 (C161W) (Fig. 2b, bottom panel), although in this latter mutant, cross-reaction with the anti-serotype 1 serum appeared to be slightly more intense than that observed for the CPS from the field serotype 14 strain. Taken together, these results demonstrate that a single amino acid substitution (W161C or C161W) in the glycosyltransferase CpsK is sufficient to effect serotype switching in each pair of serotypes (2 and 1/2, and 14 and 1).

**Table 1:** Results of the coagglutination test.

Strain	Tested antisera			Interpretation
	Anti-serotype 1	Anti-serotype 2	Anti-serotype 14	
SS2	−	+		Serotype 2
SS2to1/2	+	+		Serotype 1/2
SS1/2	+	+		Serotype 1/2
SS1/2to2	−	+		Serotype 2
SS14	−		+	Serotype 14
SS14to1	+		+	Serotype 1
SS1	+		+	Serotype 1
SS1to14	−		+	Serotype 14

### The W191 CpsK variant adds a Gal residue to the CPS repeating unit; the C191 CpsK variant adds a GalNAc residue instead

**Figure 3:**
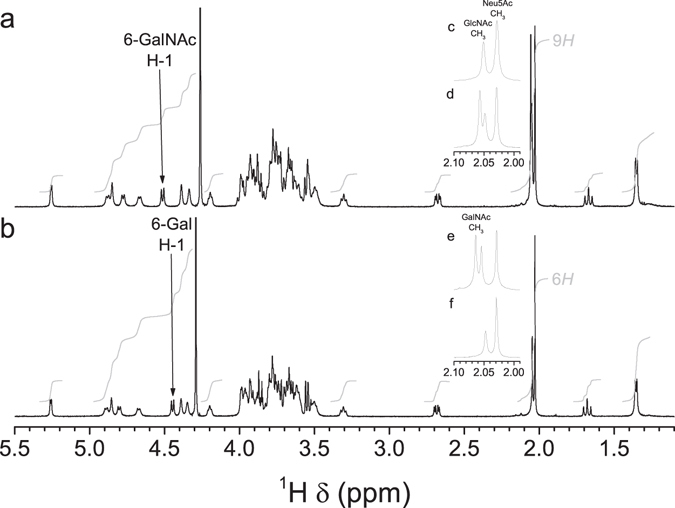
1D ^1^H NMR spectra of CPS preparations from serotypes 2 and 1/2 in 33 mM phosphate pD 8.0 in D_2_O. (**a**,**b**) Full spectrum. (**c–f**) Expansion of the methyl region. (**a**,**d**) SS2to1/2 mutant, 500 MHz, 77 °C. (**b**,**f**) SS1/2to2 mutant, 500 MHz, 75 °C. (**c**) Serotype 2 field strain, 600 MHz, 50 °C^17^. (**e**) Serotype 1/2 field strain, 700 MHz, 42 °C^19^. Specific characteristics of the ^1^H NMR spectrum due to the presence of GalNAc in the native serotype 1/2 CPS were also found in the spectrum of the CPS from the corresponding mutant expressing serotype 1/2 CPS. Conversely, the spectrum of the CPS from the mutant expressing serotype 2 CPS, as well as that of the native serotype 2 CPS, lacked the signal attributed to *N*-acetyl of GalNAc.

**Figure 4:**
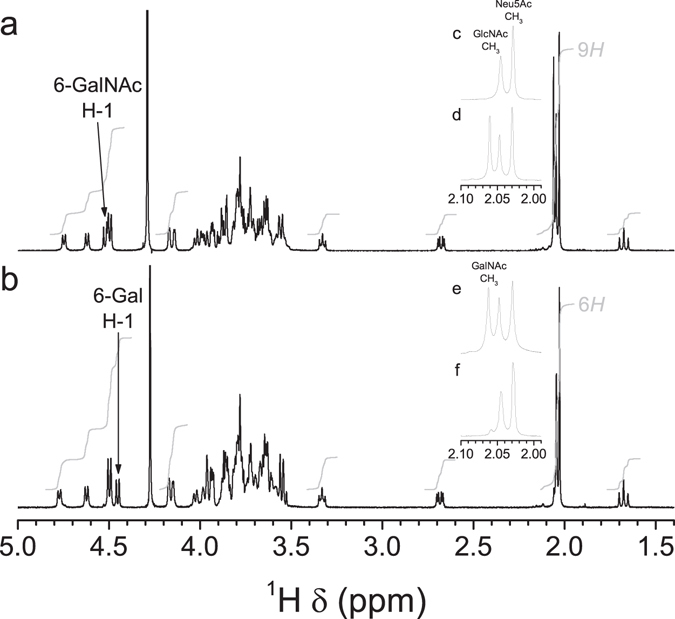
1D ^1^H NMR spectra of CPS preparations from serotypes 14 and 1 in 33 mM phosphate pD 8.0 in D_2_O. (**a**,**b**) Full spectrum. (**c–f**) Expansion of the methyl region. (**a**,**d**) SS14to1 mutant, 500 MHz, 75 °C. (**b**,**f**) SS1to14 mutant, 500 MHz, 77 °C. (**c**) Serotype 14 field strain, 500 MHz, 77 °C^18^. (**e**) Serotype 1 field strain, 700 MHz, 70 °C^19^. Specific characteristics of the ^1^H NMR spectrum due to the presence of GalNAc in the native serotype 1 CPS were also found in the spectrum of the CPS from the corresponding mutant expressing serotype 1 CPS. Conversely, the spectrum of the CPS from the mutant expressing serotype 14 CPS, as well as that of the native serotype 14 CPS, lacked the signal attributed to *N*-acetyl of GalNAc.

To further test the hypothesis that a single amino acid substitution confers CpsK polymorphic variants with different sugar substrate predilection, we next performed nuclear magnetic resonance (NMR) analysis of purified CPSs obtained from each pair of field strains and derivative serotype switching mutants using previously described protocols^17–19^. The analysis revealed one noticeable additional methyl group signal (δ 2.06) from GalNAc in the one-dimensional (1D) ^1^H NMR spectrum of the CPS preparation from the SS2to1/2 mutant (Fig. 3a and d), as well as in the spectrum of the CPS preparation of the SS14to1 mutant (Fig. 4a and d), compared to CPS preparations from the parental field strains of serotypes 2 (Fig. 3c) and 14 (Fig. 4c). Inversely, this signal was absent from the spectra of CPS preparations from mutants SS1/2to2 (Fig. 3b and f) and SS1to14 (Fig. 4b and f) and present in the spectra of CPS preparations of parental field strains of serotype 1/2 (Fig. 3e) and serotype 1 (Fig. 4e). In the anomeric region, the chemical shift of H-1 of the side-chain 6-substituted residue (GalNAc or Gal) depended on the sugar identity (Figs 3a,b and 4a,b). To determine the position of the H-1–H-2 cross peak, we acquired correlation spectroscopy (COSY) spectra (Supplementary Fig. 2a to d). H-2 resonated at a much higher frequency when an *N*-acetamido moiety instead of a hydroxyl group was present on C-2: for CPSs from SS2to1/2 and SS1/2to2 mutants, the H-1/H-2 signal was found at δ 4.51/3.92 and 4.44/3.54, respectively, as opposed to δ 4.44/3.54 and 4.49/3.94 in the CPSs from parental field strains of serotypes 2 and 1/2, respectively. Similarly, for CPSs from the SS14to1 and SS1to14 mutants, the signal was found at δ 4.52/3.93 and 4.45/3.54, as opposed to δ 4.45/3.54 and 4.51/3.93 in the CPSs from parental field strains of serotypes 14 and 1, respectively^17–19^. A small shift of the anomeric proton of GlcNAc, to which GalNAc or Gal is attached, was also observed in all cases (Supplementary Fig. 2a to d). Collectively, ^1^H and COSY NMR spectra unambiguously demonstrated that the SS2to1/2 and the SS1/2to2 mutants synthesized serotypes 1/2 and 2 CPSs, respectively. Similarly, the data unequivocally demonstrated that the SS14to1 and the SS1to14 mutants synthesized serotypes 1 and 14 CPSs, respectively.

It is apparent from the previous results that CPSs expressed by field strains of serotypes 2, 14, 1/2, and 1 (henceforth defined as “native” CPSs) have the same sugar composition and repeating unit structure as those expressed by mutants SS1/2to2, SS1to14, SS2to1/2, and SS14to 1 (henceforth defined as “mutant” CPSs), respectively. To investigate whether other differences existed between native and mutant CPSs, we next performed size-exclusion chromatography coupled with multi-angle light scattering (SEC–MALS). The predicted molecular mass (*M*_w_) of the different CPS preparations were relatively similar between serotype 2 “native” CPS (435 kg/mol) and serotype 2 “mutant” CPS (prepared from strain SS1/2to2) (504 kg/mol), and between serotype 14 “native” CPS (421 kg/mol) and serotype 14 “mutant” CPS (prepared from strain SS1to14 (571 kg/mol), suggesting similar chain lengths. However, “mutant” serotype 1/2 and “mutant” serotype 1 CPSs (prepared from strains SS2to1/2 and SS14to1, respectively) appear to have reduced CPS chain lengths when compared to “native” serotype 1/2 CPS (483 vs. 709 kg/mol) or “native” serotype 1 CPS (490 vs. 741 kg/mol), respectively (Supplementary Table 1). However, it must be noted that data for “mutant” CPS were acquired by analysis of a single batch of CPS preparation per mutant strain. Further experiments are needed to confirm whether those differences in *M*w actually represent differences in CPS length.

### Three-dimensional modeling of polymorphic CpsK with either W161 or C161 is compatible with substrate predilection for Gal or GalNAc, respectively

**Figure 5:**
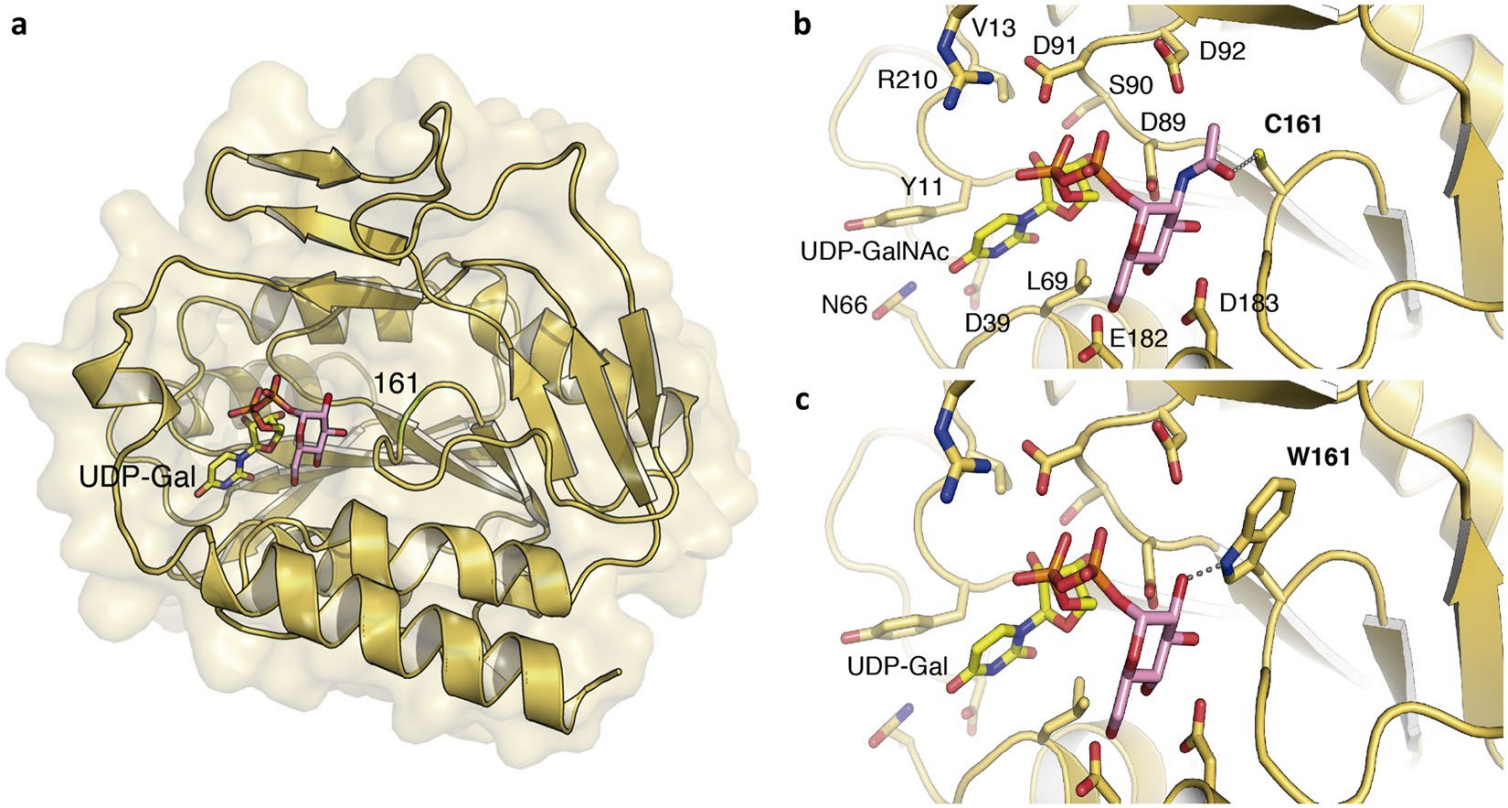
Three-Dimensional modeling of CpsK polymorphic variants. (**a**) The serotype 2 CpsK protein structure is depicted in yellow ribbon, and the position of amino acid residue 161 was colored in green and labeled. The docked substrate UDP-Gal is shown in sticks with the Gal moiety in pink. (**b**) Detailed view of the catalytic center of protein CpsK from serotype 1/2 with a cysteine residue at position 161 (C161) in complex with UDP-GalNAc as a substrate. Residues predicted to play a role in substrate binding and stabilization are depicted as capped sticks and labeled. Potential hydrogen bond between C161 and *N*-acetyl group of GalNAc is represented with a dashed grey line. (**c**) Same view as in panel a for the catalytic center of protein CpsK from serotype 2 with a tryptophan residue at position 161 (W161) in complex with UDP-Gal molecule as a substrate. Dashed grey line represents the potential hydrogen bond between W161 and the hydroxyl group of Gal.

Since the only difference between each pair of parental and mutant strains is one SNP in the *cpsK* gene, we concluded from experiments presented above that the polymorphism in amino acid 161 of CpsK is the sole factor determining which sugar residue this glycosyltransferase adds to the CPS repeating unit. To investigate substrate predilection of both polymorphic forms of CpsK in more detail, we next built a three-dimensional (3D) model for the serotype 2 CpsK protein variant (bearing W161) (Fig. 5a). CpsK belongs to the glycosyltransferase family 2 (GT2) that is a member of the clan GT-A, all of which present two tightly associated β/α/β domains that form a central eight-strands β-sheet in a Rossman-like fold. As described in the carbohydrate-active enzymes (CAZY) database (www.cazy.org)^21^, GT2 enzymes present an inverting mechanism. Members of this family are responsible, generally, for the transfer of nucleotide-diphosphate sugars to substrates such as polysaccharides and lipids. Strict conservation of the nucleotide-binding site and availability of several 3D structures complexed with different substrates allowed us to identify the localization of the saccharide moieties bound to the activated nucleotide sugar in CpsK. Residue 161 is located at the core of the catalytic center at the beginning of the last β-strand of the central β-sheet and close to the nucleotide-biding site (Fig. 5a). Our modeling analysis revealed that recognition of the nucleotide is not affected by replacement of residues at position 161. Indeed, all the residues required for recognition of the uracil group, the ribose moiety, and the pyrophosphate group are conserved in both variants of CpsK (Fig. 5 and Supplementary Fig. 3). Docking of uridine diphosphate (UDP)-Gal and UDP-GalNAc in the active sites of both CpsK variants provided a clear explanation of the potential role of residue 161 in substrate specificity. C161 in CpsK from serotype 1/2 could stabilize the GalNAc residue in the UDP-GalNAc substrate by establishing a polar interaction with the acetyl group of the sugar (Fig. 5b) (in addition to the potential H-bonds created by the conserved residues with the other oxygen atoms from the GalNAc sugar). The distance between the SH group of C161 and the carbonyl oxygen of GalNAc is in the range of a hydrogen bond formation (3.1–3.7 Å, according to the values stored in the Cambridge Structural Database). The same happens for the interaction of UDP-Gal with W161-bearing CpsK from serotype 2 (Fig. 5c). In this case, the W161 residue shapes the cavity to accommodate this smaller ligand and provides an H-bond with the O2 of Gal through the N atom of the indole ring (Fig. 5c). Interestingly, this interaction is also observed in the 3D structure of one of CpsK closest homologues, the chondroitin polymerase from *Escherichia coli*, in complex with UDP-glucuronic acid (Protein Data Bank database [PDB] code 2Z86) where one of the N atoms of the side chain of H581 makes an H-bond with the O2 of glucuronic acid (Supplementary Fig. 3b). In both cases, the predicted interactions would contribute to the specificity of each CpsK variant for their respective substrates. However, while each polymorphic form can stabilize its natural substrate (UDP-Gal and UDP-GalNAc in CpsK from serotypes 2 and 1/2, respectively), the exchange of substrates is not possible. With C161, there is enough room to accommodate the *N*-acetyl group bound to the galactosamine moiety at the catalytic groove. However, with W161, the steric hindrance generated between the *N*-acetyl group and the side chain of W161 would prevent the accommodation of UDP-GalNAc at the catalytic groove (Supplementary Fig. 4). Taken together, 3D modeling results provide an explanation to the differential galactosyltransferase and *N*-galactosaminyltransferase activities detected for CpsK W161 and C161 polymorphic variants, respectively.

### The CPSs of serotypes 2, 1/2, 14, and 1 have similar antiphagocytic properties, and serotype switching does not alter strain virulence

Although the virulence of *S. suis* is multifactorial, studies with mutant strains impaired in CPS expression have conclusively shown that the CPS plays a key role in the pathogenesis of infection of this pathogen^22–24^. However, little is known on the effect of serotype switching on the virulence of any given strain. One hypothesis is that inasmuch as the organism expresses a capsule the specific CPS type of any particular strain will not affect virulence. However, the fact that only a few serotypes (notably, serotype 2) predominate among strains isolated from diseased animals and humans (serotype 1/2 strains have never been isolated from human cases so far) might indicate that the specific type of CPS may be important *per se* in defining the virulence of the strain. To begin to differentiate between these hypotheses, we took advantage of the fact that our mutant strains were generated from parental field strains belonging to different STs with known virulence differences. Indeed, the serotype 2 parental field strain belongs to ST1, the parental serotype 1/2 field strain belongs to ST28, the serotype 14 parental field strain belongs to ST6, and the serotype 1 parental field strain belongs to ST1. Thus, with the exception of the spurious mutation in *gatB* noted above in mutant strain SS1to14, each pair of parental and mutant strains are truly serotype variants that differ by only one SNP genome-wide, and the differences in virulence between pairs of parental field and mutant strains may only result from their different CPSs.

**Figure 6:**
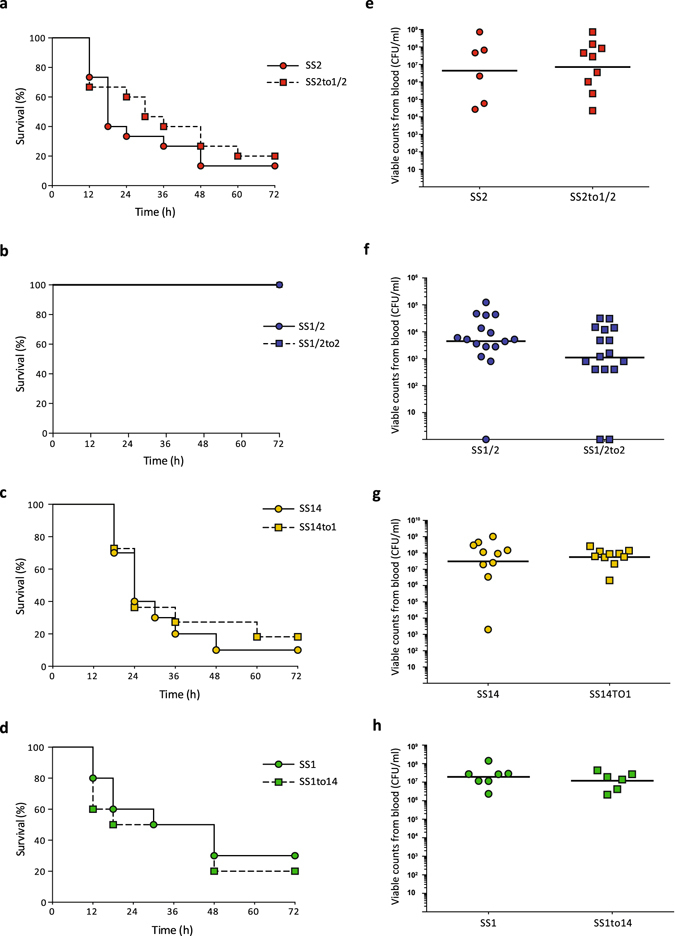
Serotype switching does not impact strain virulence. (**a–d**) Survival of CD1 mice inoculated by intraperitoneal injection with either 5 × 10^7^ CFU of serotype 2 (**a**) or 1/2 (**b**) strains or with either 1 × 10^8^ CFU of serotype 14 (**c**) or 1 (**d**) strains. In all cases, Log-Rank (Mantel-Cox) test revealed no significant differences in survival rates between the field parental strains and derivative mutants. All control animals injected with vehicle (Todd Hewitt Broth) survived the trial (data not shown for simplicity). (**e–h**) Bacterial load in blood was evaluated in all groups by drawing 5 μl of blood from the tail vein of mice followed by plating and enumeration (see methods). (**e**) Serotype 2 field strain and derived mutant SS2to1/2. (**f**) Serotype 1/2 field strain and derived mutant SS1/2to2. (**g**) Serotype 14 field strain and derived mutant SS14to1. (**h**) Serotype 1 field strain and derived mutant SS1to14. No significant differences in bacterial load were observed between parental strains and their corresponding mutants (Mann-Whitney Rank Sum test, P < 0.05).

Virulence assay results using a validated murine model of infection^25^ showed that the virulence of the SS2to1/2 mutant strain (expressing serotype 1/2 CPS) was virtually identical to that of the parental serotype 2 field strain (*P* = 0.4510). Indeed, both strains caused at least 80% mouse mortality after 3 days post-infection, as expected for highly virulent ST1 strains (Fig. 6a). Mice in both groups had high bacteremia after 24 h post-infection (*P* = 0.8917) (Fig. 6e). In addition, mice infected with either strain showed severe clinical signs such as depression, swollen eyes, rough coat hair, and lethargy. On the other hand, the serotype 1/2 parental strain and its derivative mutant strain SS1/2to2 (expressing serotype 2 CPS) were of low virulence, consistent with previously reported low virulence of ST28 strains^26^. No mortality was recorded in either group (*P* = 1.0000) after 3 days (Fig. 6b), and mice showed only mild clinical signs of infection and low bacteremia in general (*P* = 0.1116) (Fig. 6f). Similarly, no virulence differences were observed between the serotype 14 field strain (ST6 genetic background) and mutant SS14to1 (*P* = 0.6273). Indeed, both groups showed more than 80% mortality, high bacteremia (*P* = 0.6842), and severe clinical signs of infection (Fig. 6c and g). Finally, the virulent serotype 1 ST1 field strain showed high virulence with 70% mortality after 3 days post infection, and mutant SS1to14 induced similar mortality (80%) (*P* = 0.6419) (Fig. 6d). Similarly to other tested ST1 strains, mice infected with either strain showed high bacteremia (*P* = 0.5583) (Fig. 6h) and severe clinical signs.

**Figure 7:**
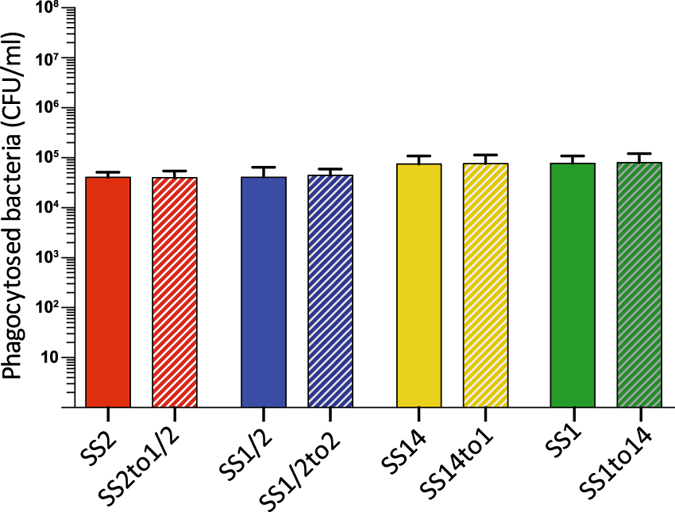
The antiphagocytic properties afforded to *S. suis* by CPS types 2, 1/2, 14, and 1 appear similar under *in vitro* conditions. Parental strains and isogenic mutants (1 × 10^7^ CFU/ml) were incubated for 60 min with J774 macrophages (multiplicity of infection = 100) in the presence of 50% murine serum. Results represent the mean (CFU/ml) + SEM of four independent experiments. Statistical analyses using the Student’s t-test showed no significant differences in the number of internalized bacteria between strains.

Next, we investigated *in vitro* the antiphagocytic properties of the different four *S. suis* CPSs by means of phagocytosis assays. Results showed that, independently of CPS type and strain genetic background, all strains were similarly internalized by murine macrophages in the presence (Fig. 7) (P = 0.9662 for SS2 and SS2to1/2, *P* = 0.8873 for SS1/2 and SS1/2to2*, P* = 0.9874 for SS14 and SS14to1 and *P* = 0.9639 for SS1 and SS1to14) or absence (data not shown) of serum. Thus, substitution of Gal by GalNAc, or *vice versa*, does not significantly alter the antiphagocytic properties of the tested CPSs. Taking the *in vitro* and *in vivo* virulence assays together, we conclude that the different CPSs possess similar antiphagocytic properties, that serotype switching does not impact the virulence of *S. suis* strains that share a similar genetic background, at least for the four serotypes tested here, and that the virulence arsenal particular to the specific genetic background of a given strain is more likely to influence its virulence.

## Discussion

Integrated systems biology approaches combining sequencing of multiple genomes of closely related organisms, in combination with animal infection models and relevant *in vitro* approaches, have been instrumental in recognizing the key contribution of small genetic changes such as SNPs and short insertion/deletions to the virulence, phenotypic characteristics, and other important biological traits of strains of several bacterial species^27–29^. Here, we show that a single amino acid polymorphism in the glycosyltransferase CpsK leads to enzyme variants with differential substrate predilection for Gal and GalNAc, defining the sugar residue added to the CPS repeating unit and thus determining four *S. suis* serotypes. Specifically, we demonstrate that for serotype pairs 2 and 1/2, and 14 and 1, a CpsK variant with W161 results in strains that are serotypes 2 and 14, while a CpsK variant with C161 results in strains that are serotypes 1/2 and 1. Our findings provide a definitive molecular explanation to intriguing previous results showing that strains of serotypes 2 and 1/2, and strains of serotypes 14 and 1, have *cps* loci with identical gene content, but their CPS structures differ between members of each pair, namely by the presence of either a Gal or a GalNAc as the CPS side-chain sugar residue bearing sialic acid^16–19^. However, NMR is our sole source of structural data, and further studies are needed to elucidate the impact, if any, of the polymorphism on other important CPS characteristics such as the number of synthesized CPS chains and their lengths.

Glycosyltransferases are a large family of proteins that are ubiquitous in bacteria and eukaryotes^30^. Despite the large number of sequence families that have been defined, structural analysis has shown that all but a few glycosyltransferases possess GT-A or GT-B folds. The catalytic domain of the GT-A-fold enzymes can be viewed as a single domain composed by two closely abutting β/α/β Rossmann domains. The Rossmann fold is found in proteins that bind nucleotides and is responsible for binding the nucleotide sugar donor substrate. With only one exception, GT-A enzymes have been found to possess a DXD motif and are metal-ion-dependent glycosyltransferases. The GT-B-fold enzymes possess also two Rossmann domains but separated by a cleft that binds the acceptor. The carboxy-terminal domain is primarily responsible for binding the nucleotide sugar donor substrate. Unlike enzymes that contain the GT-A fold, the GT-B glycosyltransferases are metal-ion independent and do not possess a DXD motif. In this study, the targeted SNP corresponding to amino acid 161 of CpsK protein is located within the glycosyltransferase functional domain. 3D modeling using relevant available crystal structures clearly suggests that the amino acid substitution at position 161 of *S. suis* CpsK leads to conformational and functional changes that permit the enzyme to select between either Gal or GalNAc. A SNP in the gene encoding the glycosyltransferase *wcrL* of *Streptococcus pneumoniae* has been shown to be responsible for the CPS differences observed between serotypes 11A and 11D of that species^31^. However, WcrL variants were shown to have bi-specificity for both Gal and GalNAc, and the resulting CPS differences were due to variable capsular Gal/GalNAc repeat unit ratio^31^. In contrast, our data indicate that *S. suis* CpsK variants are monospecific and incorporate either Gal (W161) or GalNAc (C161).

The CPS plays a key role in *S. suis* virulence. TEM showed that all four isolallelic mutant strains generated here were as encapsulated as their respective parental strains of serotypes 2, 1/2, 14 and 1. Most previous studies have only investigated the impact of abolishing CPS expression on the virulence of the organism^23, 32^. These types of studies cannot differentiate whether a specific CPS composition is important for the virulence of a strain. For example, work on *S. pneumoniae* has shown that specific CPS types endow the strains with differential ability to avoid complement deposition and modulate the virulence of the strain in murine infection models^33, 34^. Previous studies that have compared the virulence of *S*. *suis* strains expressing different CPS types have, for the most part, used strains with dissimilar genetic background or whose genetic backgrounds were not known^35^. Here, the use of isoallelic mutants and both *in vitro* and *in vivo* infection models permitted us to conclude that the CPS composition plays an unnoticeable role in the virulence of *S. suis* strains of serotypes 2, 1/2, 14, and 1. Indeed, a highly virulent parental ST1 serotype 2 strain was as virulent in mice as its isoallelic mutant expressing serotype 1/2 CPS, while the low virulence of an ST28 serotype 1/2 remained essentially unchanged in its isoallelic mutant expressing type 2 CPS. Similarly, highly virulent ST1 serotype 1 strain expressing serotype 14 CPS and virulent ST6 serotype 14 strain expressing serotype 1 CPS were as virulent as their parental strains. Moreover, we observed no differences in the antiphagocytic properties of CPS 2, 1/2, 14, and 1. One limitation of our study in comparison with the abovementioned work on *S. pneumoniae* is that we evaluated CPS types that differ only by one sugar, i.e., CPS structural changes are relatively minor and may thus not significantly impact virulence. Additionally, it can be hypothesized that the CPSs tested here may possess similar virulence-related properties. Indeed, the cross-reactions between serotypes 2 and 1/2 and serotypes 14 and 1 CPSs in the coagglutination test^16^ support the idea that these different CPSs elicit partially overlapping immune responses from the host^19^. *S. suis* strains of serotypes 2 and 14 have caused human disease, while, to our knowledge, strains of serotypes 1 and 1/2 have not^1^. Our results suggest that this differential ability to cause disease in the human host is unlikely to be related to the different compositions and structures of the CPSs of strains of the two serotype pairs.

Small genetic changes such as short insertion/deletions and, particularly, SNPs are key contributors to the genetic diversity of bacterial pathogens^36^. Their impact on bacterial phenotypic traits, including virulence, is only beginning to be uncovered. Here, we show that a single amino acid polymorphism at position 161 of the glycosyltransferase CpsK defines the enzyme specificity for either Gal or GalNAc, and that incorporation of either sugar residue into the CPS repeating unit by polymorphic CpsK is the crucial event in the differentiation between *S. suis* serotypes 2 and 1/2 and between serotypes 14 and 1. Our findings solve a 3-decade long dilemma about the nature of serotyping cross-reactions in *S. suis* serotypes 2, 1/2, 14, and 1, and extend our understanding of how small genetic changes influence bacterial traits and pathogenesis of infection.

## Methods

### Bacterial strains and culture conditions

Bacterial strains and plasmids used in this study are listed in Supplementary Table 2. Well-characterized clinical isolates of serotype 2 (strain P1/7)^37^, serotype 14 (strain DAN13730)^18^, serotype 1/2 (strain 2651)^19^ and serotype 1 (strain 1659834) were used. *S. suis* field strains and mutants were grown in Todd-Hewitt broth (THB) or agar (THA) (Becton-Dickinson, Franklin Lakes, NJ, USA) at 37 °C. *E. coli* strains were grown in Luria-Bertani broth or agar at 37 °C. When needed, antibiotics (Sigma-Aldrich, Oakville, ON, Canada) were added to the culture media at the following concentrations: for *S. suis*, spectinomycin at 100 μg/ml; for *E. coli*, kanamycin and spectinomycin at 50 μg/ml and ampicillin at 100 μg/ml.

### DNA manipulations

*S. suis* genomic DNA was purified using InstaGene Matrix (BioRad, Mississauga, ON, Canada). Oligonucleotide primers (listed in Supplementary Table 3) were from Integrated DNA Technologies (Coralville, IA, USA). Plasmid preparations were performed using the QIAprep Spin Miniprep kit (Qiagen, Toronto, ON, Canada). Restriction enzymes and DNA-modifying enzymes were purchased from ThermoFisher (Waltham, MA, USA) and used according to the manufacturers’ recommendations. PCR reactions were carried out with iProof high-fidelity DNA polymerase (BioRad) or with Taq DNA polymerase (Qiagen). Amplification products were purified using the QIAquick PCR purification kit (Qiagen) and sequenced with an ABI 310 automated DNA sequencer using the ABI PRISM dye terminator cycle sequencing kit v3 (ThermoFisher).

### Mutant generation

SNP replacements in gene *cpsK* were performed by allelic exchange. PCR amplicons were generated using specific primers and cloned into plasmid pCR2.1 (ThermoFisher), extracted using EcoRI, and subcloned into the thermosensitive *E. coli-S. suis* shuttle vector pSET4s^38^ previously digested with EcoRI, giving rise to replacement vectors p4cpskG483T and p4cpskT483G. These vectors were then electroporated into recipient *S. suis* strains using a Biorad Gene Pulser Xcell apparatus (BioRad) under specific conditions (12.5 kV/cm, 200 Ω, and 25 μF). Isoallelic mutants were isolated as previously described^39^. Sanger sequencing confirmed adequate replacement of nucleotide 483 of *cpsK* genes. Whole-genome sequencing using Illumina MiSeq technology of all parental and mutant strains, and polymorphism identification were performed as previously described^21^.

### Serotyping

Serotyping was performed by coagglutination as previously described^10^. Results were deemed positive when a strong reaction was obtained within 1 min or less. Dot blot assays were used to confirm the CPS antigenicity of constructed mutants using highly purified CPS preparations, as previously described^19^.

### Transmission electron microscopy

TEM was carried as previously described^23^. Unless otherwise indicated, chemicals were from Sigma-Aldrich. Briefly, bacteria were grown to mid-logarithmic phase and washed in 0.1 M cacodylate buffer, pH 7.3. Rabbit antisera (150 μl) directed against the different CPS types were used for CPS stabilization. Next, cells were immobilized in 4% (w/v) agar in 0.1 M cacodylate buffer pH 7.3 (Canemco & Marivac, Canton de Gore, QC, Canada). Prefixation was performed adding 0.1 M cacodylate buffer, pH 7.3, containing 0.5% (v/v) glutaraldehyde and 0.15% (w/v) ruthenium red for 30 min. Fixation was performed for 2 h at room temperature with 0.1 M cacodylate buffer, pH 7.3, containing 5% (v/v) glutaraldehyde and 0.05% (w/v) ruthenium red. Post-fixation was carried out with 2% (v/v) osmium tetroxide in water at 4 °C for 16 h. Samples were washed with water every 20 min for 2 h to remove osmium tetroxide and dehydrated in increasing graded series of acetone. Specimens were then washed twice in propylene oxide and embedded in Spurr low-viscosity resin (Electron Microscopy Sciences, Hatfield, PA, USA). Thin sections were post-stained with uranyl acetate and lead citrate and examined with a transmission electron microscope at 80 kV (Hitachi model HT7770, Chiyoda, Tokyo, Japan).

### Purification and physicochemical characterization of CPS

*S. suis* strains were grown in 150 ml of THB at 37 °C for 16 h, diluted to 6 l in fresh THB, and grown overnight. The cells were pelleted by centrifugation at 10,000 × *g* for 40 min, suspended by repeated pipetting in 33 mM phosphate-buffered saline (PBS), pH 8.0, and chilled. The CPSs were then purified as previously described^17^. Purified CPSs were characterized by SEC–MALS, and *M*_w_ of each CPS was determined as previously described^18, 40^.

### NMR spectroscopy

CPSs were exchanged in phosphate buffer, pD 8.0, in D_2_O (99.9 atom % D), freeze dried, and dissolved in D_2_O (99.96 atom % D) to a final concentration of 33 mM. NMR spectra were acquired on polysaccharide samples at concentrations of 0.4–1.3%. ^1^H chemical shifts δ in ppm were referenced to internal deuterated 2,2-dimethyl-2-silapentane-5-sulfonate at δ 0 as recommended by Wishart *et al*.^41^. Spectra were acquired at 11.75 T on a Bruker Avance 500 spectrometer equipped with a 5-mm triple resonance TBI probe with ^1^H, ^13^C, and ^109^Ag–^31^P channels at 75–77 °C or at 16.45 T on a Bruker Avance 700 spectrometer with a 5-mm cryoprobe with ^1^H and ^13^C channels at 42 °C using standard Bruker pulse sequences at the Centre régional de résonance magnétique nucléaire, Department of Chemistry, University of Montreal. Conventional 1D ^1^H spectra were acquired with 30° pulses. The gradient-enhanced two-dimensional (ge-2D) COSY spectrum was acquired in magnitude mode using 45° or 90° pulses with or without purge pulses, respectively. Spectra were processed off-line with the software package SpinWorks 4.2.0.0 available at http://home.cc.umanitoba.ca/~wolowiec/spinworks/ For 1D spectra, 32–40 K complex data points were acquired and processed by exponential multiplication with a line-broadening factor equal to the digital resolution, zero filling, complex Fourier transform, phase correction, and fifth-order polynomial baseline correction. Zhu-Bax forward–backward linear prediction with 16 coefficients was systematically applied to 2D processing in the *f*_1_ dimension^42^.

### Modeling methods

Protein similarity searches were carried out with BLASTP https://blast.ncbi.nlm.nih.gov using the *S. suis* serotype 2 CpsK amino acid sequence against the PDB database. Three available 3D structures showed high identity with the catalytic module of CpsK and were identified as (i) a putative glycosyltransferase from *Streptococcus parasanguinis* (GalT1, PDB code 5hea, identity 39.13% covering 98% sequence), (ii) a putative glycosyltransferase from *Bacteroides fragilis* (PDB code 3bcv, identity 35.6% covering 88% sequence), and (iii) the chondroitin polymerase from *E. coli* strain K4 in complex with UDP (PDB code 2z87, identity 28% covering 52% sequence). We selected PDB 5hea and used it as a template to build a structural model for *S. suis* CpsK with either W161 or C161. Structural models for both variants were generated independently using the Swiss-Model server^43^. For modeling the interaction with ligands UDP-Gal and UDP-GalNAc, we used the two available 3D structures complexed with substrates that showed the highest identity with *S. suis* CpsK: (i) chondroitin polymerase from *E. coli* strain K4 (K4CP) complexed with UDP-glucuronic acid and UDP (PDB code 2z86 and 2z87, identity 23.22%) and (ii) the human UDP-GalNAc:polypeptide α-*N*-acetylgalactosaminyltransferase^44^ (pp-GalNAc-T10, PDB code 2D7i, identity 18%). UDP-Gal and UDP-GalNAc PDBs were built using the electronic Ligand Builder eLBOW implemented in Phenix^45^. The generated substrates were structurally superimposed using the UDP-glucuronic acid present in PDB 2z86 as a template.

### Experimental animal infection

All experiments involving animals were conducted in accordance with the guidelines and policies of the Canadian Council on Animal Care and the principles set forth in the Guide for the Care and the Use of Laboratory Animals by the Animal Welfare Committee of the University of Montreal, and approved by the latter Committee (Protocol RECH-1570). A well-standardized *S. suis* murine model of infection was used^25^. A total of 80 six-week-old female CD1 mice (Charles River Laboratories, Wilmington, MA, USA) were acclimated to standard laboratory conditions. On the day of the experiment, mice were assigned randomly to 8 groups of 10 mice each. Each group was inoculated by intraperitoneal injection of 1 ml of bacterial suspensions of either one field strain or its derivative mutant expressing a switched serotype. Bacterial inocula were 5 × 10^7^ colony forming units (CFU) for serotypes 2 and 1/2 and corresponding mutants or 1 × 10^8^ CFU for serotypes 14 and 1 and corresponding mutants. These inocula were chosen based on preliminary trials carried out with parental strains and a reduced number of animals (data not shown). Mice were monitored at least three times a day for mortality and clinical signs of systemic disease, such as depression, swollen eyes, rough coat hair, and lethargy. To evaluate bacteremia, blood samples were collected from the tail vein at 12, 24, 48, and 72 h post-infection, plated onto THA using an Autoplate 4000 Automated Spiral Plater (Spiral Biotech, Norwood, MA, USA) and bacterial colonies enumerated after incubation at 37 °C for 16 h.

### Phagocytosis assay

Phagocytosis assays were performed using the murine macrophage cell line J774A.1 (ATCC TIB 67) maintained and cultured as previously described^46^. For bacterial phagocytosis, 48 h cell cultures were scraped, washed twice with phosphate-buffered saline (PBS), pH 7.4, and resuspended in antibiotic-free medium at 1 × 10^5^ cells/ml. Cell suspension was then distributed into 24-well tissue culture plates (1 ml/well) and incubated for 3 h to allow cell adhesion. The cell culture medium was removed and cells were infected by adding 250 μl of a 4 × 10^7^ CFU/ml bacterial suspension in culture medium (without antibiotics) and 250 μl of mouse serum (from C56BL/6 mice and stored at −80 °C), to obtain a ratio of 100 bacteria per cell. The infected cells were incubated for 60 min at 37 °C with 5% CO_2_ to allow phagocytosis. Assay conditions were chosen based on preliminary studies (data not shown). After incubation, cells were washed with warm PBS and incubated for 1 h in medium containing 5 μg/ml penicillin G (Sigma-Aldrich) and 100 μg/ml gentamicin (ThermoFisher) to kill extracellular bacteria as previously described^46^. After antibiotic treatment, cells were washed and lysed with 1 ml of sterile distilled water. After vigorous pipetting to ensure complete cell lysis, viable intracellular bacterial counts were determined by plating serial dilutions onto THA using an Autoplate 4000 Automated Spiral Plater. Each test was repeated four times in independent experiments, and the number of CFU recovered per well (mean ± SEM) was determined.

### Statistical analysis

All data are expressed as mean ± SEM. *In vitro* data were analyzed for significance using the Student’s t-test. Normality was previously verified in order to use Student’s t-test. Log-Rank (Mantel-Cox) test was used to analyze survival rates between parental field strains and derivative serotype switching mutants in animal infection assays. Statistical analyses for bacteremia were calculated using the Mann-Whitney Rank Sum test. A *P* value < 0.05 was used as a threshold for significance.

## Electronic supplementary material

Supplementary information
