# Supplementary material for: A single amino acid polymorphism in the glycosyltransferase CpsK defines four Streptococcus suis serotypes
Source: Sci Rep. 2017 Jun 22;7:4066. doi: 10.1038/s41598-017-04403-3 (PMC5481389; doi:10.1038/s41598-017-04403-3)
Supplement: Supplementary file 1 — Supplementary information [file 41598_2017_4403_MOESM1_ESM.pdf]

Supplementary information for manuscript:

A single amino acid polymorphism in the glycosyltransferase CpsK defines four *Streptococcus suis* serotypes

David Roy[1], Taryn B.T. Athey[2], Jean-Philippe Auger[1], Guillaume Goyette-Desjardins[1], Marie-Rose Van Calsteren[1,3], Daisuke Takamatsu[4,5], Masatoshi Okura[4], Sarah Teatero[2], Martín Alcorlo[6], Juan A. Hermoso[6], Mariela Segura[1], Marcelo Gottschalk[1] & Nahuel Fittipaldi[2,7]*

[1]Swine and Poultry Infectious Diseases Research Centre, Faculty of Veterinary Medicine, University of Montreal, Saint-Hyacinthe, QC J2S 2M2, Canada.

[2]Public Health Ontario, Toronto Laboratory, Toronto, ON M5G 1M1, Canada.

[3]Saint-Hyacinthe Research and Development Centre, Agriculture and Agri-Food Canada, Saint-Hyacinthe, QC J2S 8E3, Canada.

[4]Division of Bacterial and Parasitic Diseases, National Institute of Animal Health, National Agriculture and Food Research Organization, Tsukuba, Ibaraki, 305-0856, Japan.

[5]The United Graduate School of Veterinary Sciences, Gifu University, Gifu, Gifu, 501-1193, Japan

[6]Department of Crystallography and Structural Biology, Instituto de Química-Física "Rocasolano", Consejo Superior de Investigaciones Científicas, 28006 Madrid, Spain.

[7]Department of Laboratory Medicine and Pathobiology, University of Toronto, Toronto, ON M5S 1A8, Canada

This supplementary file contains 3 Supplementary Tables and 4 Supplementary Figures.

Supplementary Table 1. Average yields for CPS purification of the different *S. suis* field and mutant strains and CPS molecular weights.

Strain	Serotype	Average yield ± SD[a] (number of replicates)	M_w (kg/mol)	Reference
SS2	2	46.3 ± 19.1 (13) [b]	435 ± 32.1 (4) [b]	[1]
SS2to1/2	1/2	51.0 (1)	483 (1)	This work
SS1/2	1/2	73.5 ± 2.1 (2)	709 (1)	[2]
SS1/2to2	2	44.6 (1)	504 (1)	This work
SS14	14	30.1 ± 14.8 (11) [b]	421 ± 117 (3) [b]	[3]
SS14to1	1	28.2 (1)	490 (1)	This work
SS1	1	37.5 ± 9.2 (2)	741 (1)	[2]
SS1to14	14	32.4 (1)	571 (1)	This work

[a] Expressed in mg of CPS/6L of culture

[b] Means calculated from published and unpublished laboratory data.

Supplementary Table 2. Bacterial strains and plasmids used in this study.

Strains/Plasmid	General Characteristics	Source/Reference
Escherichia coli		
TOP 10	F-*mrcA* Δ(*mrr-hsd*RMS-*mcr*BC)*ϕ80 lacZ*Δ*M5* Δ*lac*X74 *rec*A1 *ara*D139 Δ(*ara-leu*) 7697 *gal*U *gal*K *rps*L (StrR) *end*A1 *nup*G	ThermoFisher
Streptococcus suis		
SS2	Strain P1/7; ST1, serotype 2 strain isolated from a swine clinical case of infection in the United Kingdom. Identified as SS2 in this study.	[4]
SS1/2	Strain 2651; ST28 serotype 1/2 strain isolated from a swine clinical case of infection in Denmark. Identified as SS1/2 in this study.	[5]
SS14	Strain DAN13730; ST6 serotype 14 strain isolated from a human case in The Netherlands. Identified as SS14 in this study.	[6]
SS1	Strain 1659834; ST1 serotype 1 strain isolated from a swine clinical case of infection in Canada. Identified as SS1 in this study.	This work
SS2to1/2	Isoallelic *cpsK* mutant of serotype 2 strain P1/7 carrying G483T mutation predicted to result in W161C substitution in mature CpsK	This work

SS1/2to2	Isoallelic *cpsK* mutant of serotype 1/2 strain 2651 carrying T483G mutation predicted to result in C161W substitution in mature CpsK	This work
SS14to1	Isoallelic *cpsK* mutant of serotype 14 strain DAN13730 carrying G483T mutation predicted to result in W161C substitution in mature CpsK.	This work
SS1to14	Isoallelic *cpsK* mutant of serotype 1 strain 1659834 carrying T483G mutation predicted to result in C161W substitution in mature CpsK.	This work
Plasmids		
pCR2.1	Ap^r, Km^r, *oriR*(f1) MCS *oriR* (ColE1)	ThermoFisher
pSET-4s	Thermosensitive vector for allelic replacement. Replication functions of pG+host3, MCS *oriR* pUC19 *lacZ* Sp^R	[7]
p4cpskG483T	pSET-4s carrying the construct for allelic replacement in strain P1/7 (SS2) and strain DAN13730 (SS14)	This work
p4cpskT483G	pSET-4s carrying the construct for allelic replacement in strain 2651 (SS1/2) and strain 1659834 (SS1)	This work

Supplementary Table 3. Oligonucleotide primers used in this study.

Name	Sequence (5' – 3')	Constructs
sero1-2_ID1	GCGGTATCTTTAATAGCCCTTG	p4cpskT483G
sero14_ID1	AGATACTATACGTTGGCAAG	p4cpskG483T
sero1-2_ID4	CATAGTAACTCCCAACTCCCTG	p4cpskT483G, p4cpskG483T
sero14_ID5	GAGATTCTTCTGGTGAATGACG	p4cpskT483G, p4cpskG483T
sero1-2_ID8	CCCCGTTTTCAGAAAGACAC	p4cpskT483G, p4cpskG483T

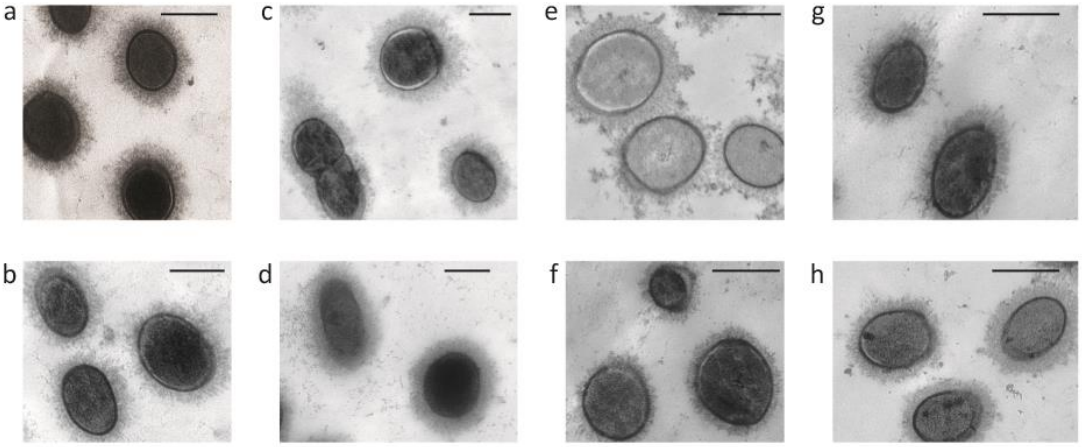

Supplementary Figure 1. Transmission electron micrographs showing capsular polysaccharide (CPS) expression by *S. suis* field strains and derivative capsular switch mutants. The CPS was stabilized with polyclonal antibodies directed against the CPS (see methods). **a,** Serotype 2 field strain. **b,** Mutant strain SS2to 1/2. **c,** Serotype 1/2 field strain. **d,** Mutant strain SS1/2to2. **e,** Serotype 14 field strain. **f,** Mutant strain SS14to1. **g,** Serotype 1 field strain. **h,** Mutant strain SS1to14. No noticeable differences were observed between field strains and corresponding derivative mutant strains in CPS expression. Bar: 500 nm.

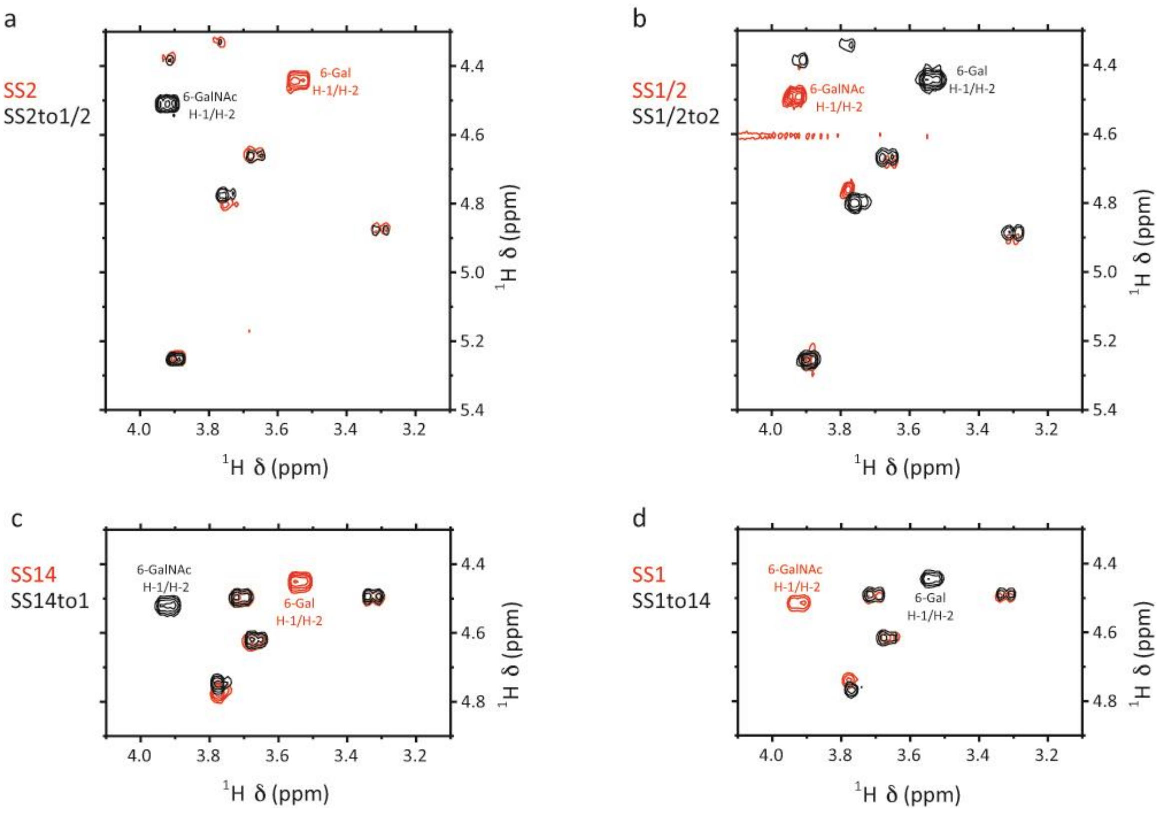

Supplementary Figure 2. Portion of the ge-2D NMR COSY spectrum of *S. suis* CPSs in 33 mM phosphate pD 8.0 in D_2O. In each panel, signals from the mutant and native CPSs are depicted in black and red, respectively. **a,** Mutant SS2to1/2. 500 MHz, 77°C. 512 increments of 1 K complex data points were acquired in magnitude mode with a digital resolution of 3.9 Hz/point in the t_2 dimension and 7.8 Hz/point in the t_1 dimension; the t_2 dimension was processed by multiplication with an unshifted sine bell window function and Fourier transform, and the t_1 dimension was processed by Zhu-Bax linear prediction to 1024 points, multiplication with an unshifted sine bell window function, Fourier transform, and magnitude calculation. Serotype 2 field strain. 500 MHz, 75°C. 512 increments of 1 K complex data points were acquired in magnitude mode with a digital resolution of 4.6 Hz/point in the t_2 dimension and 9.2 Hz/point in the t_1 dimension; processing was as above. **b,** Mutant SS1/2to2. 500 MHz, 75 °C. 512 increments of 1 K complex data points were acquired in magnitude mode with a digital resolution of 4.9

Hz/point in the t_2 dimension and 9.8 Hz/point in the t_1 dimension; processing was as described in **a**. Serotype 1/2 field strain, 700 MHz, 42°C. 512 increments of 2 K complex data points were acquired in magnitude mode with a digital resolution of 3.4 Hz/point in the t_2 dimension and 13.7 Hz/point in the t_1 dimension; processing was as described in **a**. **c,** Mutant strain SS14to1, 500 MHz, 75 °C. Serotype 14 field strain, 500 MHz, 77°C. Acquisition and processing were as described in **b**. **d,** Mutant strain SS1to14, 500 MHz, 77°C. Serotype 1 field strain, 500 MHz, 65°C. Acquisition and processing were as described in **b**.

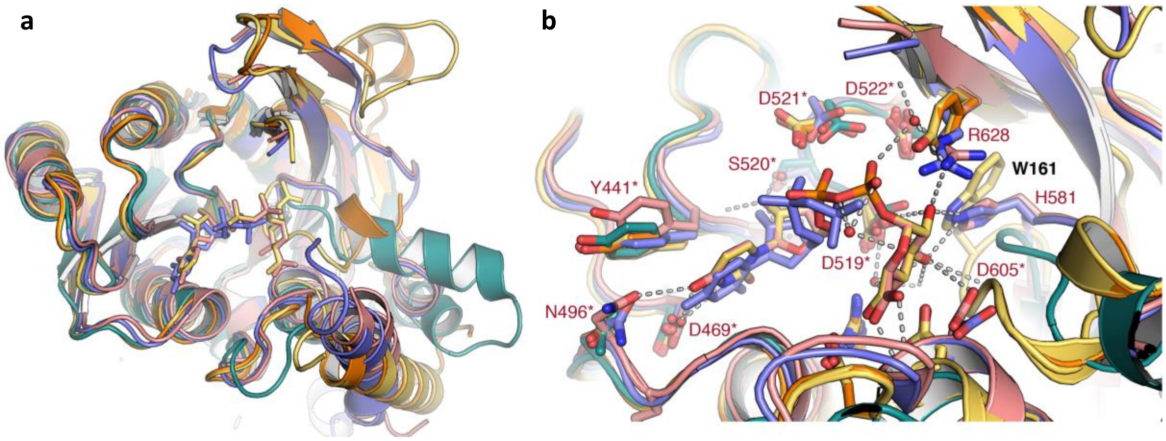

Supplementary Figure 3. Structural and substrate-binding conservation in closest homologues of CpsK from *S. suis*. a, Structural superposition of the model of CpsK from *S. suis* in complex with UDP-GalNAc (yellow ribbon and sticks) and the putative glycosyltransferase (GalT1) from *Streptococcus parasanguinis* (PDB code 5hec, orange ribbon), the chondroitin polymerase from *Escherichia coli* strain K4 in complex with UDP (PDB code 2z87, blue ribbon and sticks), the chondroitin polymerase from *Escherichia coli* strain K4 complexed with UDP-GlcUA (PDB code 2z86, pink ribbon and sticks) and the glycosyltransferase from *Bacteroides fragilis* (PDB code 3bcv, green ribbon). All these structures share a common fold with key residues interacting with the dinucleotide virtually overlapping. The estimated RMSD value for the Cα backbone of the catalytic core between all the structures was less than 1.14 Å. **b,** Detailed view of the nucleotide-binding site in CpsK serotype 2 (yellow ribbon) in complex with UDP-GalNAc (yellow sticks) and its closest homologues (color code as in panel **a**). Residues involved in substrate binding are represented as capped sticks. Interactions between chondroitin polymerase from *E. coli* strain K4 and UDP-GlcUA (PDB code 2z86) are represented as dashed lines. Residues involved in the interaction are labeled. Asterisk indicates if the residue is conserved in CpsK. Trp161 in CpsK is depicted as yellow capped sticks to stress potential conservation of the interaction with O2 of Gal ring observed for His581 with glucuronic acid.

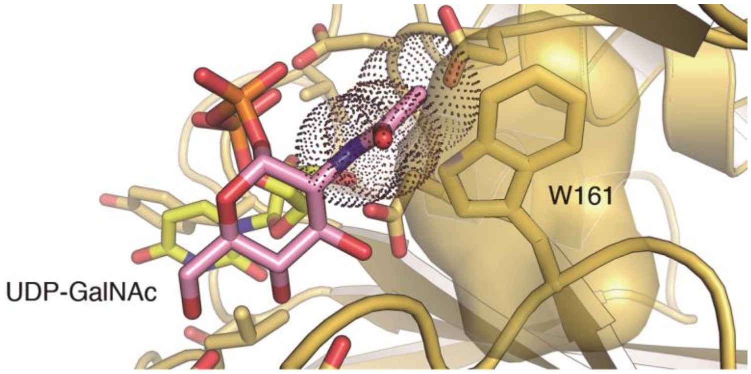

Supplementary Figure 4. Steric hindrance between tryptophan 161 and the *N*-acetyl group of UDP-GalNAc. Tryptophan residue is labeled and depicted in sticks with its molecular surface shown in yellow. The UDP-GalNAc is represented in sticks with the UDP moiety colored in yellow for C atoms and the GalNAc moiety colored in pink for C atoms. The Van der Waals radius of the *N*-acetyl group is shown as dots representation. The presence of bulky Trp residue at position 161 would prevent binding of GalNAc at the active site of CpsK from serotype 2.

References

1. Van Calsteren MR, Gagnon F, Lacouture S, Fittipaldi N, Gottschalk M. Structure determination of *Streptococcus suis* serotype 2 capsular polysaccharide. *Biochem Cell Biol* **88**, 513-525 (2010).

2. Van Calsteren MR*, et al.* Explaining the serological characteristics of *Streptococcus suis* serotypes 1 and 1/2 from their capsular polysaccharide structure and biosynthesis. *J Biol Chem*, **291**, 8387-8398 (2016).

3. Van Calsteren MR*, et al.* Structure determination of *Streptococcus suis* serotype 14 capsular polysaccharide. *Biochem Cell Biol* **91**, 49-58 (2013).

4. Slater JD, Allen AG, May JP, Bolitho S, Lindsay H, Maskell DJ. Mutagenesis of *Streptococcus equi* and *Streptococcus suis* by transposon *Tn*917. *Vet Microbiol* **93**, 197-206 (2003).

5. Jacques M, Gottschalk M, Foiry B, Higgins R. Ultrastructural study of surface components of *Streptococcus suis*. *J Bacteriol* **172**, 2833-2838 (1990).

6. Gottschalk M, Higgins R, Jacques M, Mittal KR, Henrichsen J. Description of 14 new capsular types of *Streptococcus suis*. *J Clin Microbiol* **27**, 2633-2636 (1989).

7. Takamatsu D, Osaki M, Sekizaki T. Thermosensitive suicide vectors for gene replacement in *Streptococcus suis*. *Plasmid* **46**, 140-148 (2001).
